# Excitotoxins, Mitochondrial and Redox Disturbances in Multiple Sclerosis

**DOI:** 10.3390/ijms18020353

**Published:** 2017-02-08

**Authors:** Cecilia Rajda, Dániel Pukoli, Zsuzsanna Bende, Zsófia Majláth, László Vécsei

**Affiliations:** 1 Department of Neurology, University of Szeged, 6725 Szeged, Hungary; rajda.cecilia@med.u-szeged.hu (C.R.); pukoli.daniel@med.u-szeged.hu (D.P.); bendzsu@gmail.com (Z.B.); majlathzsofia@gmail.com (Z.M.); 2 Department of Neurology, Vaszary Kolos Hospital, 2500 Esztergom, Hungary; 3 MTA-SZTE Neuroscience Research Group, 6725 Szeged, Hungary

**Keywords:** biomarker, excitotoxin, glutamate, oxidative stress, mitochondria, multiple sclerosis, neurodegeneration

## Abstract

Multiple sclerosis (MS) is a chronic inflammatory disease of the central nervous system (CNS). There is increasing evidence that MS is not only characterized by immune mediated inflammatory reactions, but also by neurodegenerative processes. There is cumulating evidence that neurodegenerative processes, for example mitochondrial dysfunction, oxidative stress, and glutamate (Glu) excitotoxicity, seem to play an important role in the pathogenesis of MS. The alteration of mitochondrial homeostasis leads to the formation of excitotoxins and redox disturbances. Mitochondrial dysfunction (energy disposal failure, apoptosis, etc.), redox disturbances (oxidative stress and enhanced reactive oxygen and nitrogen species production), and excitotoxicity (Glu mediated toxicity) may play an important role in the progression of the disease, causing axonal and neuronal damage. This review focuses on the mechanisms of mitochondrial dysfunction (including mitochondrial DNA (mtDNA) defects and mitochondrial structural/functional changes), oxidative stress (including reactive oxygen and nitric species), and excitotoxicity that are involved in MS and also discusses the potential targets and tools for therapeutic approaches in the future.

## 1. Introduction

Multiple Sclerosis (MS) is an inflammatory central nervous system (CNS) disorder associated with demyelination and neurodegeneration, which cause temporary or permanent neurological symptoms [1]. Previous opinions suggested that the initial process of the disease was inflammation, followed later by neurodegenerative mechanisms, including oxidative stress, neuronal and oligodendrocyte (ODC) apoptosis. Recent research has provided data that neurodegeneration does not follow inflammation, but that it is present simultaneously and related [2,3]. One of the connecting links between inflammation and neurodegenerative mechanisms is glutamate excitotoxicity [4,5]. In MS, involvement of both white and gray matter is well known. Peculiar pathological patterns of white matter lesions are infiltration of immune cells (lymphocytes, macrophages, and microglia), Ig and complement deposition, ODC death, demyelination, axonal loss, and astrogliosis. Their occurrences in a white matter lesion depend on the lesion’s activity and pathologic profile [2,6,7,8]. Gray matter lesions have been associated with clinical disability and cognitive impairment [8,9,10]. They are characterized by an intact blood-brain barrier (BBB), the absence of lymphocyte infiltration, antibody deposits, and complements, therefore, grey matter lesions are considered mainly “non-inflammatory” [8,9,11,12,13]. Several reports suggest that both inflammatory and neurodegenerative processes contribute to the pathology of grey matter but their relationship is unexplained. According to a relatively new theory (the so-termed “inside-out” theory), a primary cytodegeneration of ODCs and/or neurons would appear initially years before the first symptoms, and the autoimmune inflammatory reaction would direct against the autoantigens released during cellular degeneration [3,9]. More plausible hypothesis is that adaptive immune processes provoke white and grey matter damage, and when pathological changes like axon damage and axonal loss have accessed a serious level, neurodegeneration takes over from the inflammatory course [9].

Extensive neuroimaging, immunohistochemical [8] and pathological studies are searching for the answers about whether the gray and white matter lesions are affected by the same mechanism. One hypothesis presumes retrograde degeneration secondary to the white matter lesion, but there are evidences of demyelination-independent grey matter damages in experimental autoimmune encephalomyelitis (EAE)/MS [9,14].

The chronic inflammatory process present in MS results in elevated levels of reactive oxygen species, which may lead to mitochondrial damage and consequently an energy deficit in neurons [15]. An energy deficit and chronic demyelination lead to several ion channel dysfunctions. Alterations in Glu homeostasis may result in excessive calcium influx to the cells, which may cause excitotoxic neuronal damage [15].

## 2. Excitotoxicity in Multiple Sclerosis

Glutamate (Glu) is one of the principal excitatory neurotransmitters in the CNS. All types of cells, including neurons and glial cells (oligodendrocytes, astrocytes, microglia) use it to communicate with each other [4]. Its level in the CNS is almost a thousand-fold higher than other neurotransmitters and it can rise another 55-fold [5]. Under normal conditions, the presynaptic vesicles release Glu into the synaptic cleft. Because of its toxicity, it must be cleared away quickly before it accumulates. Astrocytes are mainly involved in the reuptake of Glu via Glu transporters and in its conversion to glutamine, a non-excitatory amino acid, via the glutamine synthetase pathway [16]. In the Glu-glutamine cycle, the main enzymes involved are glutaminase and glutamine-synthetase [17,18]. Glu and its metabolism are present in white matter (WM) too. ODCs have glutamine synthetase activity [19] and produce Glu dehydrogenase [6,20]. Microglia, the main source of extracellular Glu uses glutaminase to produce it from glutamine [21].

The concept of Glu excitotoxicity in the CNS was established by Olney in 1969 [22], who injected monosodium Glu in newborn mice and observed acute neuronal necrosis. In excitotoxicity, excessive stimulation of Glu-binding receptors by extracellular Glu dispatches the intracellular cascade mechanisms—which lead to cell death.

Alterations in Glu homeostasis were observed both in MS and EAE. Stover et al. (1997) [23] was the first to provide evidence for the participation of Glu excitotoxicity in MS: they found a significantly elevated Glu level in the cerebrospinal fluid (CSF) of MS patients in the acute phase of the disease (data are shown in Table 1). Sarchielli and colleagues measured elevated Glu levels in MS patients’ CSF, in different stages of disease (see Table 2) [24]. In 2005, Srinivasan et al. showed increased Glu levels with MR spectroscopy in acute lesion of MS patients [25]. There was a correlation between the Glu level and the extension of axonal injury [26]. In EAE, aberrations in the expression of Glu transporters, receptors or metabolizing enzymes were detected which suggested the role of excitotoxicity in this model of MS. Pathological changes like axonal damage, demyelination, and ODC loss were observed which are exhibitive of excitotoxicity. The use of GluR antagonists could suspend these effects and cause disease amelioration [6,27,28,29,30,31].

Previous knowledge about glutamate excitotoxicity applied principally to grey matter (GM) pathology. However Glu impacts not only in GM, but WM too [8], so attention has turned toward WM processes, because the injuries in MS involve mainly this part of the CNS [4,32]. The main cell type found here are the ODCs [27]. In MS, the chief WM pathological changes include ODC death and axonal degeneration [7], in which excitotoxicity has high priority [6]. In MS/EAE, there are pathologic changes in almost all parts of Glu homeostasis [27] caused by endogenic (genetic) or exogenic (environmental) triggers [5,27]. Because of these alterations, the rapid elimination of Glu is not possible. Excessive accumulation of this neurotransmitter is toxic to the cells [4,33]. The GM pathology in MS/EAE received little attention until recently, when extensive histological studies, proteomic investigations, MRI imaging techniques, and animal model research showed evidence of an independent (or at least partly independent) pathological change in brain cortical regions in both MS and EAE [8,32,34]. Besides axonal damage and retrograde neuronal loss, early damage in synaptic functioning with synaptic loss, called synaptopathy occurs. It has a long-lasting impact on motor and cognitive function of MS patients. Synaptopathy and neuronal damage, in addition to axonal injury, are primarily responsible for patients’ disability [9,34]. The exact processes causing synaptopathy are not known, but Glu excitotoxicity might have a substantial role in it [34].

Various kind of molecular and cell mechanisms are responsible for the extensive Glu release (Table 3). They involve elevated Glu production by different types of CNS and activated immune cells, altered transporter function, glutamate receptor overexpression, and enzyme defects both in WM and GM [6].

The first and most important sources of extracellular Glu are the activated microglia/macrophage cells and leukocytes [5,35]. At the onset of the disease microglia activation is an important protective mechanism (the cells potentiate tissue repair and disposal of misfolded proteins), but in the chronic phase of MS/EAE it will be deleterious and subserve neuronal death [5]. Microglia cells are activated in all subtypes of MS [43] and they release a great amount of Glu to induce inflammation [35]. Microglia have very low or no glutamine synthetase activity [44], so they require glutamine uptake from the extracellular space to produce Glu. These cells have high glutaminase activity [21]. They discharge Glu into the extracellular space via gap junction-like hemipores [36] and system x_c_^−^ cystine/Glu antiporters (x_c_^−^) [37,38], which transport cystine into the cell converting it to Glu while elevating the extracellular Glu level. The cell will produce glutathione, a potent antioxidative agent from cystine [45].

To worsen the situation, microglia (and in a lesser manner astrocytes too) release tumor necrosis factor alpha (TNF-α), a proinflammatory cytokine, which plays an important role in microglia-mediated Glu emission [21]. It enhances Glu excitotoxicity by decreasing the expression of EAATs and detoxifying enzymes in glial cells, which lowers the possibility of reuptake of further Glu and increases ionotropic GluRs’ localization in synapses [5,46,47]. In an in vitro study, TNF-α upregulated the glutaminase 1 isoform in human neuronal cell culture [48].

Recent studies have revealed that astrocytes can not only take up Glu, but also emit it in a Ca^2+^-dependent and/or -independent manner, i.e., with x_c_^−^ antiporter, or with Glu transporters (excitatory amino acid transporters = EAATs), if they change transport direction [4,49]. With a self-perpetuating mechanism, Glu can increase its self-production via metabotropic Glu receptors in these cells [39]. TNF-α can promote Glu delivery by binding to astrocyte TNFR-1 receptors [40]. Aggravating the impact of excitotoxicity, the demyelinated axons may emit additional amounts of Glu. Ectopically distributed Ca^2+^ channels will appear on the injured axon membrane and, due to pathological Ca^2+^ influx, Glu will be liberated from vesicles [41]. Na^+^-dependent Glu transporters will activate in the reverse direction which results in Glu ejection into the extracellular space [50]. With this Glu release, the damaged axon turns against itself and will enhance further injury by intensifying glutamate excitotoxicity in a vicious cycle.

In the CNS, to prevent the toxic effects of Glu, fast reuptake of the neurotransmitter from the synaptic cleft is required [4,16]. Reuptake is assisted by the electrochemical gradient of Glu across the plasma membrane and is accomplished by Glu transporter proteins, i.e., EAATs. These transporter proteins are expressed mainly by astrocytes, but they can also be found on ODC, neuron, and microglia membranes [16]. In WM, ODCs are primarily responsible for the removal of extracellular Glu [20]. Presently five different families of EAATs are known (EAAT1–EAAT5). They vary in Na^+^ and/or K^+^ coupling abilities. Their names differ according to whether the transporter is found in human or in other mammals (see Table 4).

Under pathological circumstances like MS, compensatory EAAT overexpression guarantees the riddance of Glu, as found in EAE [51]. Dysfunction of the transporters leads to extracellular Glu accumulation and consecutive excitotoxicity. In MS lesions, decrease in EAAT2 levels extends from the center to the edge of the lesion, while the EAAT1 and -3 levels are normal [6]. In vitro and in vivo experiments proved that glial transporter-1 (GLT-1) hinders ODC death and axonal degeneration. This effect could be suspended by using α-amino-3-hydroxy-5-methyl-4-isoxazolepropionic acid receptor (AMPA) antagonists [52]. Other studies give controversial results about the role of the EAATs in MS/EAE pathology and their role in eliminating Glu [53,54]—on the one hand their downregulation is presumed to cause elevated Glu levels, but on the other hand a lot of data are available for their overexpression [4,20,55]. More research is required to clarify their role.

In human ODCs and in EAE brain, glutamine synthetase and Glu dehydrogenase activity are reduced [6,20]. In chronic lesions, minimal function of these enzymes was revealed in astrocytes and microglia [6]. This dysfunction of Glu metabolizing enzymes, which inhibits the OCDs from getting rid of Glu, enhances the damage caused by Glu excitotoxicity long after the inflammation ceases [4]. The cause of the enzyme defect in ODC is unknown; cytokines and oxidative stress may take part in it. Glutamine synthetase is highly susceptible to oxidative injury [4]. However, the glutaminase enzyme is upregulated both in active MS lesions and in EAE [42,56,57,58] which leads to elevated Glu synthesis. As the greatest amount of Glu is expressed by microglia, inhibiting microglial glutaminase could lower extracellular Glu levels and attenuate excitotoxic effects. Unfortunately, up to now, there has been no potent glutaminase inhibitor available. In 2014 Thomas and colleagues developed a cell-based microglia activation assay for the evaluation of glutamate-levels and microglia glutaminase activity, and to demonstrate its expedience in investigating the effects of glutaminase inhibitors. In their study, they found decreased Glu levels in the presence of the glutaminase inhibitors bis-2-(5-phenylacetimido-1,2,4-thiadiazol-2-yl)ethyl sulfide (BPTES), JHU-198 and JHU-212. The same decrease was noticed in the absence of glutamine. They suggest the use of this microglia assay in developing potent glutaminase inhibitors [21].

Glu exerts its effect by binding glutamate receptors (GluRs) which initiate a signaling cascade inside the cell resulting in apoptosis [4]. The receptors are classified into two groups: ionotropic and metabotropic. Ionotropic receptors are voltage-gated ion channels, which allow Ca^2+^ or K^+^ to enter the cells and initiate downstream signaling [5], while metabotropic Glu receptors are atypical G-protein associated receptors.

Glutamate ionotropic receptors (iGluRs) are divided into NMDA-, AMPA- and kainate receptor subtypes. They are named after their favorable agonist, *N*-methyl-d- aspartate (NMDA), α-amino-3-hydroxy-5-methyl-4-isoxazolepropionicacid (AMPA) and kainic acid (kainate). 

Metabotropic receptors are categorized into three groups, mGLuR I, mGLuR II, and mGluR III. These receptors can be found on neurons, ODCs, and axons too [42]. They commence second messenger pathways, for example phospholipase C, phosphoinositide 3 kinase/retrovirus AK thymoma/mTOR (PI3K/AKT/mTOR), and mitogen-activated protein kinase (MAPK) signaling [5]. They are classified into three group (mGluR-I, -II, and -III) [4]. Details can be seen in Table 5. 

In CNS, NMDA receptors are found mainly in neurons, making them the most sensitive for glutamate excitotoxicity [4]. In circumstances of normal synaptic transmission, the activated receptor allows Ca^2+^ through its ion channel only for a short time, then the channel becomes blocked by Mg^2+^, preventing the cell from excessive Ca^2+^ overload [59]. In pathological conditions like MS, this mechanism diminishes, allowing Ca^2+^ influx into the cell [4].

In 2005 Káradottír found functional NMDA receptors on ODC cells in EAE rat brain [60]. These receptors were localized on myelin-forming projections. Their role in ODC is presumed to help myelination. Where only little cytoplasm is present in the projections, low levels of ion influx cause significant ion-concentration elevation with more severe damage (swelling and demyelinating) [60]. NMDA receptors have greater Glu-affinity than α-amino-3-hydroxy-5-methyl-4-isoxazolepropionic acid receptors (AMPARs). In neurodegenerative disorders (like MS/EAE), where prolonged Glu release is less common, *N*-methyl-d-aspartate receptors (NMDARs) are more likely to be activated than AMPARs, and NMDARs localized on ODCs are less sensitive to Mg^2+^ blockades, thus are much more susceptible to glutamate excitotoxicity [60]. These results raise the question whether NMDARs exist on human ODCs or not. In 2016, Livesey et al. examined human ODC progenitor cells in all stages of development. They could not demonstrate any response to NMDA, but that effect may be a result of the maintenance conditions of the cell cultures [61]. Further research is needed to disclose the importance of these kinds of receptors in MS/EAE pathology.

In the human and rodent CNS, AMPARs are found mainly on glial cells including ODCs. They are responsible for high-speed excitatory neurotransmission [4]. Ion permeability of these receptors depends on subunit arrangement, leaking Na^+^, K^+^, and/or Ca^2+^ ions. GluA2 subunit is highly relevant, its presence in the receptor composition means a loss of Ca^2+^-permeability [59]. In animal studies there is much evidence to suggest the role of AMPARs in EAE pathology with lowered ODC viability and axonal damage.

Matute et al. (1997) demonstrated that activated AMPARs lower the ODCs’ viability in rat ODC cell culture, but this effect can be diminished by the extraction of Ca^2+^ from the cell medium [62]. By blocking the receptors with the antagonist NBQX (2,3-dihydroxy-6-nitro-7-sulfamoyl-benzo(f)uinoxaline-2,3-dione), the symptoms of EAE improved with the reduction of ODC death and axonal damage [28,63]. In vivo GLT-1 block enhanced ODC death and axonal injury, but the effect could be suspended by AMPA-antagonists [52]. Axonal damage is induced by neuronal AMPARs [64]. However, Wosik et al. (2004) demonstrated low or missing AMPA/kainate receptor expression in in vitro human cell culture and in human brain sections [65]. In their opinion, whilst rodent ODCs produce high levels of AMPARs and have great vulnerability to Glu excitotoxicity, human cells exposed to long-continued dosages of agonists do not have this property and they resist AMPA/kainate-mediated excitotoxicity. If they are present in vivo, it goes against the presumption that Glu excitotoxicity plays a part in the initial damage of myelin-forming ODCs in EAE and MS lesions. In response to these findings Livesey et al. (2016) [61] examined similarities and differences between rodent and human ODC membrane components, receptors, and features in different stages of development. They revealed AMPAR expression in all studied cells including mature ODCs. Their results showed corresponding features and regulation of AMPARs in rodent and human cells. Another question to be answered regards the components of AMPA receptors expressed on OCDs. The presence of the GluA2 subunit results in impermeability to Ca^2+^. Immunostaining examination revealed a predomination of GluA2-free, Ca^2+^ permeable AMPA receptors in ODCs found in MS lesions [42]. A study with GluA3 knock-out mice confirmed this result [66]. These findings support the relevancy of AMPA receptors in the pathology of MS/EAE.

In an animal model of MS, Centonze and colleagues examined synaptic changes. They found that excessive Glu accumulation activated AMPAR but not NMDAR, which led to increased excitatory postsynaptic currents (EPSCs) and altered synaptic function [14,67].

Kainate receptors localized on pre- and postsynaptic membranes take part in synaptic signal transduction. Generally it is associated with AMPA [4]. In WM, functional kainate receptors are expressed on the surface of ODCs [4]. The role of these receptors in MS/EAE was first proposed by Matute in 1998, who injected kainate into the rat optic nerve and observed MS-like lesions [68]. Alberdi et al. (2006) revealed that in humans ODCs exposed to kainate and AMPA antagonist GYKI53655, intracellular Ca^2+^ overloads caused cell death [69]. Activation of kainate receptors by Glu induces sensitivity to complement toxicity in ODCs, which could be another important factor in demyelination in MS/EAE. Kainate receptors can be found on axons too, resulting in axonal degeneration by Ca^2+^ influx into the axon when they are activated [64]. These receptors are only partially involved in the pathology of MS/EAE and cannot initiate glutamate excitotoxicity per se. Examinations with selective kainate antagonists (UBP296, ACET, NS 3763 and topiramate) could not influence the course of the MS/EAE [4].

Evidence exists that activation of mGlu-I receptors enhances the function of NMDA receptors on neurons [70]. These receptors are also localized on ODC precursor cells, which play a beneficial role in maintaining glutathione levels and protect against oxidative stress. Thus these receptors have dual roles in the pathology of MS/EAE by damaging axons in lesional neurons and promoting remyelination by enlarging the viability of ODC precursors [4]. Because they are not expressed on mature ODCs, they cannot take part in primary glutamate excitotoxicity leading to cell death [4].

Glu has controversial impacts on CNS immune activity depending on which mGluRs exerts its effect. It can be immunosuppressive or increase the expression of proinflammatory cytokines. Glu can reduce the neurotoxicity of microglia via mGlu-III receptors or augment it through mGlu-II receptor activation and consecutive TNF-α release by activated microglia [8]. The immunomodulatory effects of mGlu-III receptors may be used to find novel therapies. Cinnabarinic acid, a partial agonist of mGlu4 (a group III metabotropic GluR) is protective in EAE. This agent is an endogenous metabolite of tryptophan (TRY) produced in the kynurenine pathway (KP) of TRY metabolism [4,71]. In addition, cinnabarinic acid influences neuroinflammation, by shifting T-cell differentiation towards the Th17 cell population, which are responsible for immune tolerance and may protect against EAE. Thus cinnabarinic acid is a link between the CNS and the immune system [71].

When Glu binds to its ionotropic receptors, high volumes of Ca^2+^ stream into the cell which leads to cell death via a cascade mechanism. Most of these processes are less known or hypothetical and include the production of free radicals, dysfunctions in mitochondrial operation and the activation of proapoptotic pathways. Excessive extracellular Glu generates overstimulation of Glu-receptors, which is the main factor for intracellular oxidative stress [4]. Beside Ca^2+^, Na^+^ will overload the cells which open Na^+^/Ca^2+^ exchangers and voltage-gated Ca^2+^ channels resulting in more Ca^2+^ influx [27]. Ca^2+^-binding proteins could buffer the effect of excitotoxicity. Unfortunately ODCs do not express them, which makes the cells more susceptible to the impact of Glu [27]. Elevated Ca^2+^ levels provoke nitric-oxide (NO) production, activation of Ca^2+^-sensitive proteases and mitochondrial injury. Proapoptotic mechanisms activate proteases (caspases and calpains) [4]. Calpains induce DNA fragmentation. Their inhibitors protect neurons from NMDA-mediated excitotoxic injuries, curiously without incorporating normal receptor functions like learning or memory progresses, and may be examined as possible novel therapeutic agents in MS [4].

### 2.1. The Role of Kynurenines in Glutamate Excitotoxicity in MS/EAE

**Figure 1:**
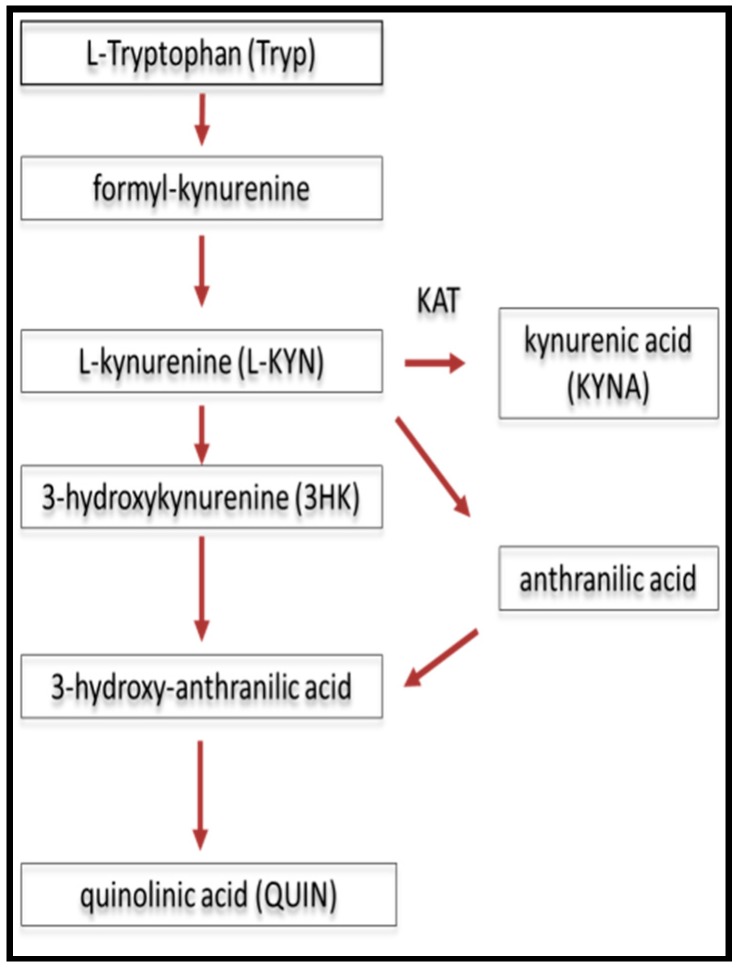
The metabolism of tryptophane: the kynurenine pathway. The red arrows show the direction of the metabolism. KAT: kynurenine–aminotranspherase (adapted from Bohár et al., 2015 [72]).

In the pathology of different neurodegenerative disorders, the metabolism of endogenous TRY to kynurenic acid (KYNA) and/or quinolinic acid (QUIN) has received intensified attention because of its dual behavior of being neuroprotective or neurotoxic [71,72,73,74,75,76,77,78]. The steps of TRY metabolism are shown in Figure 1.

KYNA, which is an endogenous antagonist of iGluRs like NMDAR, has neuroprotective attributes. Low concentrations of KYNA facilitate AMPA receptors, while increased levels interfere with them [71,77,79,80]. It can inhibit presynaptic Glu release via α7-nicotinic-acetylcholine-receptors even at low concentrations, which makes it a potent neuroprotective agent [71]. KYNA expressed by astrocytes can counterbalance the neurotoxin-producing effect of the microglia at the site of local injury [81]. During the course of MS, KYNA levels change several times. It increases in the acute phase and later, with the progression of the disease, gradually decreases because of alterations in the KP. This effect indicates the possible neuroprotective role of KYNA in neurodegenerative processes [71].

Microglial cells produce and liberate the excitotoxin NMDA-agonist QUIN [81]. In EAE rodents and in MS patients pathologically high levels of this agent were demonstrated [82]. In opposition to KYNA, QUIN facilitates neurotoxic effects by raising extracellular Glu levels through inhibiting reuptake into astrocytes and releasing Glu from neurons. It is responsible for lipid peroxidation and oxidative stress too [71]. Increased local levels of QUIN are able to participate in demyelination in EAE and possibly MS [81]. Apoptosis of different kinds of CNS cells (ODCs, neurons, and astrocytes) can be mediated by QUIN exposure in EAE [83].

### 2.2. Blood-Brain Barrier Dysfunction

The function of the BBB is to maintain and protect the special micro-environment of the CNS by ensuring the intricate molecular interactions between neurons and glial cells. In the development of MS/EAE pathology, the evolution of BBB-dysfunction is fundamental and recent studies indicate that Glu has a significant role in this. Polymorphonuclear leukocytes in CNS circulation are able to release Glu by inflammatory processes, which induces a disruption of the BBB via mGluRs [84]. Through the compromised barrier further Glu molecules leak from the serum into the CNS, augmenting excitotoxicity [5]. NMDA and kainate receptors are expressed on cerebral endothelial cells inducing more BBB injury [42,84]. On the abluminal side, EAAT1, -2, -3 can be found which eliminate superabundant Glu from the extracellular space, thereby lowering the impact of excitotoxic damages [4]. 

## 3. Mitochondrial Disturbances in EAE and MS

Increasing evidence suggests that mitochondrial dysfunction plays an important role in the neurodegenerative processes, occurring most prominently in progressive MS. These processes involve mitochondrial DNA damage, abnormal mitochondrial gene expression, insufficient mitochondrial enzyme activity, and faulty DNA repair mechanisms [85].

Our current knowledge suggests that mitochondria are genetically independent organelles that can be found in every single eukaryotic cell, possessing their own DNA. They play a very important role in providing energy to cells by synthesizing adenosine triphosphate (ATP), moreover, they play a role in the metabolism of fatty acids and in programmed cell death (apoptosis) [86,87,88,89]. The mitochondrial respiratory chain can be found on the inner mitochondrial membrane, consisting of four complexes (complex I–IV), the fifth complex contributes directly to ATP synthesis [87,88,90]. These complexes are built of multiple subunits, most parts are proteins coded by mtDNA, and only complex II is encoded by nuclear DNA [89,91]. Neurons are highly dependent on oxidative energy metabolism. The greatest amount of ATP is produced during oxidative phosphorylation. In this process, large amounts of reactive oxygen and nitrogen species (ROS and RNS, respectively) are formed that are harmful to the cell. Production of cellular antioxidants serves as a countermeasure against this process [92,93]. This process stays in a highly sensitive balance. In the specific case when ROS and RNS synthesis exceeds antioxidant synthesis it results in oxidative stress and cell components are damaged in the cell [90,91,94,95,96,97]. Mitochondrial dysfunction results in a decrease in ATP synthesis, impaired Ca^2+^ content, and increased ROS and RNS at the same time [98]. Due to increased lipid peroxidation as a result of elevated ROS levels, membrane injuries occur, secondary failures accumulate in mitochondrial DNA (mtDNA) (as secondary de novo mutations). Aging is thought to be accelerated by mitochondrial genome alterations, and the decline of energy production, although ROS production increases further.

Mitochondrial damage in MS was found to play an important role in the progression of the disease [99,100,101]. The 3-step hypothesis describes a compensatory axonal response, pre-progression of mtDNA deletions, which is still a reversible phase, and finally an irreversible phase. Axonal transport deficits were discovered early in the disease course with focal axonal degeneration [102,103]. The axonal energy failure leads to synaptic atrophy, but not loss [104,105]. Using electron microscopy, significant damage among mitochondria and microtubuli was seen, furthermore, calcium-mediated damage could be verified, resulting in chronically demyelinated axons [106,107]. Cyclophylin D (CyPD), a regulator of the mitochondrial permeability transition pore (mPTP), plays an important role in cell death due to calcium mediation and oxidative stress [108,109]. Mice lacking CyPD were found to be more resistant to oxidative stress, and axonal degeneration also occurred at a lower rate [110]. Leber’s hereditary optic neuropathy (LHON) is caused by mtDNA mutations, although other studies indicate that development of severe optic neuritis and mutations of mtDNA may have causal correlation [102,111,112,113]. Mitochondrial dysfunction was hypothesized to occur as a result of axonal degeneration in the white matter of MS lesions. Multiple studies have supported the same data [99]. In a recent study, acute mitochondrial damage was found in experimental inflammatory lesions in EAE, which resulted in focal axonal degeneration [102]. Similar changes have been found in post mortem MS lesions. A correlation between severity of inflammation and levels of ROS and RNS, produced by macrophages and microglia, has also been found in EAE and MS lesions [114,115]. It has been verified that following ROS detoxification by free radical scavengers, mitochondrial damage and focal axonal degeneration occurred at a lower rate [102]. Mitochondrial dysfunction was shown not only in WM lesions, but also in the gray matter. A comprehensive study verified a decline in activity of oxidative phosphorylation complexes I, III, and IV and deletions of mtDNA in non-myelinated post mortem motor cortex areas of MS patients [106,116]. Another study found multiple failures in several active MS lesions in complex IV protein of the mitochondrial respiratory chain [117]. An increase in the activity of complex IV proteins has been found in chronic inactive MS lesions [104]. Damaged mtDNA and decreased levels of OXPHOS complexes may be a consequence of oxidative and nitrosative stress [116,118]. 

Recent findings seem to verify the hypothesis that MS is a 2-phase disease, in which inflammation dominates at the beginning, but neurodegeneration takes over later, although, the latter can also be found in the early stages of the disease. A new study found that mitochondrial damage can precede inflammation in EAE, suggesting that mitochondrial dysfunction is primary in the disease [119]. The process of neurodegeneration and mitochondrial disturbances in MS has not yet been clarified, but the combined effects of hypoxia, superoxide, and nitric oxide may play an important role in mitochondrial dysfunction [119]. Under experimental circumstances, it has been reported in EAE, that mitochondrial dysfunction can be found in the early stage of MS disease [120]. In a recent study (based on histological evidence), the excessive production of nitric oxide by activated microglia and macrophages can be a cause of reversible conduction block, which is observed in demyelinated axons [121,122]. In EAE, it has been found that nitric oxide, superoxide, and peroxinitrite can impair mitochondrial function, thereby inhibiting mitochondrial complexes I to V, aconitase, manganese superoxide dismutase, and creatine kinase, which can lead to damage of mtDNA, lipidperoxidation and increased mitochondrial proton permeability [123,124,125].

## 4. Redox Disturbances in EAE and MS

Free radicals have a central role in several physiological and pathological processes. Both ROS and RNS originate from endogenous and exogenous sources. Mitochondria, endoplasmic reticulum, peroxisomes, phagocytic cells, and others serve as endogenous sources, and predominantly environmental factors, such as alcohol, tobacco, pollution, industrial solvents, pesticides, heavy metals, specified medicines, etc. make up the exogenous factors. Pathological conditions where free radicals are involved are diabetes, cardiovascular and respiratory diseases, cancers, Alzheimer’s and Parkinson’s disease, as well as MS. They are the product of normal cellular metabolism. Normally free radicals are involved in different physiological processes, like mitogenic response, cellular signaling pathways, redox regulation, and defense against pathogens. The molecular targets in oxidative and nitrosative stress are DNA, RNA, proteins, and lipids [126].

The CNS is particularly susceptible to damage because of its high oxygen requirement, high lipid content, and low levels of antioxidant enzymes. Antioxidants of enzymatic and nonenzymatic origin can be found in different parts of the cells (Table 6).

Blood reduced glutathione (GSH) and oxidized glutathione (GSSG) levels are an index of whole body oxidative stress [127]. Both GSH and α-tocopherol levels remain stable with aging [128]. The antioxidant enzyme superoxide dismutase (SOD) is present primarily in neurons, while GSH and glutathione peroxidase are in astrocytes [129]. In MS the source of ROS is proposed to be the activated microglia and macrophages, which induce lipid peroxidation as a key feature [130].

In EAE, significantly increased NO production, elevated malondialdehyde (MDA) levels, and reduced GSH concentration and SOD activity were found in the brain mass [131]. In the spinal cord of EAE mice significantly decreased concentrations of GSH were detected, which points to the defective expression of GSH synthesizing enzymes. Beside this, impaired Nrf2 regulation was also found [132]. Treating EAE animals with α-tocopherol resulted in both amelioration of disease activity and progression. α-Tocopherol also proved to inhibit interferon-γ production leading to a change in cytokine release favorably shifting the immune responses [133]. Biliverdin reductase improved the pathological and clinical signs of EAE, acting as a scavenger for bilirubin [134]. In the cuprizone animal model of MS, resveratrol promoted remyelination by increasing Olig1 expression, moreover improving balance and motor coordination, reversing cuprizone-induced demyelination and alleviating oxidative stress [135]. 3*H*-1,2-dithiole-3-thione, a compound found in vegetables, both delays disease onset and dramatically decreases disease severity in EAE [136].

In MS relapse, significantly increased GSSG levels were measured in the blood. Independent of the activity of the disease in MS patients, GSH levels were higher compared to controls. During exacerbation, elevated plasma-free SH groups, decreased levels of alpha-tocopherol and alpha-tocopherol/lipid ratio were found. Interferon-beta-1b therapy increased the level of alpha-tocopherol, but not the corrected lipid levels after two months of therapy [127]. After six months of interferon-β therapy, the earlier decreased α-tocopherol levels were normalized in the erythrocytes of MS patients, while the α-tocopherol/lipid ratios remained constant [137]. This finding was supported by another study, where, besides the increased alpha tocopherol levels during interferon-beta treatment, reduced disease activity on MRI was described [138]. Acar and colleagues found elevated levels of MDA, SOD, oxidative stress index in RR MS compared to healthy controls, while decreased NO and total antioxidative status levels were found [139]. In line with these, other studies increased levels of total glutathione, GSH, GSSG/GSH ratio and SOD in MS patients were also found [140]. RR MS patients had high MDA and glutathione peroxidase concentrations [141]. Conversely, slightly reduced SOD was reported among MS patients with reduced protein sulfhydryl (SH) groups [142]. These changes suggest increased free radical production and consumption of the scavenger molecules during the active phase of the disease. In cerebellar gray matter of the brain of MS patients, upregulated SOD1 and SOD2 enzymes were found [143].

Plasma lipid peroxidation studies failed to relate oxidative stress with disease progression [144]. Mitoxantrone is an antineoplastic drug used in active secondary progressive MS. After mitoxantrone therapy, a significant increase in the lipid peroxidation marker MDA level was found together with a significant reduction in MnSOD, catalase (CAT), and glutathione peroxidase (GSH-Px) activities in the CSF. In the serum MDA concentration increased and MnSOD activity decreased, while Cu/ZnSOD activity increased [145]. Brain GSH levels followed by MRI over 3–5 years of secondary progressive (SP) MS patients were lower compared to the control group and patients with progression had lower frontal GSH levels [146]. A 12 month long fish oil rich diet did not change the glutathione redox activity in MS patients [147]. 

Elevated CSF MDA and antioxidant activity was found in both MS and Guillain-Barre syndrome, while in the sera these values were significantly decreased [148]. LHON is a mitochondrial genetic disease that affects both the optic nerve and the retina. In patients with LHON and also in asymptomatic carriers decreased α-tocopherol/lipid ratio was found in the plasma, pointing to elevated tocopherol consumption and free radical generation [149]. A combined and constant deficiency of the reducing systems was found in two compound triose phosphate isomerase-deficient brothers with markedly decreased alpha-tocopherol, carotenoid and GSH levels in the plasma and erythrocytes [150]. In these diseases, the changes in the redox systems were similar to the alterations observed in MS.

α-Tocopherol modulates mitochondrial hydrogen peroxide formation in a dose dependent manner [151]. It is mainly regenerated from its phenoxycal radical by ubiquinol [152]. Decreased vitamin levels (α-tocopherol, ascorbic acid, β-carotene, and retinol) were found in the plasma of relapsing-remitting (RR) MS patients during relapse [153]. High dose vitamin consumption elevated the initially low levels of glutathione peroxidase enzyme activity of MS patients after five weeks [154]. In a heterogeneous MS group of 170 patients (consisting of mainly RR MS patients), increased levels of plasma uric acid, oxypurins, MDA, nitrite, nitrate levels, while decreased ascorbic acid levels were found [155].

## 5. Biomarkers in Tissue Damage of MS

There are currently no diagnostics sensitive and specific enough for this disorder, the diagnosis relies more on clinical features. Thus, emerging research is trending toward CSF biomarkers, which could provide more specific data reflecting the heterogeneity of MS [156]. A biomarker is “a characteristic that is objectively measured and evaluated as an indicator of normal biological processes, pathogenic processes or pharmacological responses to a therapeutic intervention” [157]. It is a good supplementary marker “that is intended to substitute for a clinical endpoint” [157]. They may be used not only to diagnose the disease more specifically, but also to predict its course and facilitate personalized therapy by examining treatment response or detecting an increased possibility of severe side effects [156]. With the specificity of biomarkers it would be possible to differentiate between various disorder appearances or other demyelinating diseases resembling MS [158]. Besides CSF samples, there is a great attempt to find less invasive methods too. The plasma, urine, and saliva of MS patients are being researched using MR spectroscopy techniques. The biomolecules which may be putative biomarkers reflect the different CNS mechanisms like immunological changes or degenerative alterations in the progression of MS. Thus excitotoxins and redox system molecules could serve as biomarkers. Their detailed discussion is beyond the framework of this review, we want only to highlight some important aspects about them.

In the active phase of the disease, glutamate levels increase in patients’ CSF as was shown in the above mentioned study by Sarchielli et al. The concentration of CSF Glu rose when the number of active demyelinating lesions increased in RRMS subjects [24]. With multivoxel magnetic resonance spectroscopy, Azevedo et al. found that in white matter with a normal appearance, elevated Glu levels and decreased *N*-acetyl-aspartate concentration (NAA) could predict disease progression [159]. MacMillan et al. found early and consistent alterations in the Glu and glutamine levels in CNS over two years in SPMS patients with MR spectroscopy. This method might be competent to measure disease progression over years, using the appropriate imaging techniques suggested by the authors [160].

Lately a great variety of oxidative stress enzymes, proteins, nucleic acids etc. have been examined in order to find new biomarkers. Some of these molecules are thiobarbituric acid reactive substances, advanced oxidation protein products, fructosamine [161], activated α-2-macroglobulin [162], MDA, ceramides [163], chemokine 11 (CCL11) [164], the total level of advanced protein oxidation (AOPP), and a decreased level of total thiol groups [165].

The concentration alterations of molecules participating in oxidative stress mechanisms could predict the degree of disability or disease progression. One of the studies showed that serum levels of TNF-α, IFN-γ, IL-4, IL-6, IL-10, and IL-17, albumin, ferritin, and plasma levels of AOPPs, NOx, and TRAP among others, might signal high disability measured by EDSS and are associated with different types of symptoms (pyramidal, sensory or cerebellar) [166].

Thiobarbituric acid reactive substances and advanced glycation end-products were found to be increased in the saliva of MS patients during relapses. This study showed higher levels of other oxidative stress biomarkers such as thiobarbituric acid reactive substances, advanced oxidation protein products, and fructosamine in plasma during relapses [161].

Isoprostanes (IsoP)—prostaglandin-like molecules originating from free radical-catalyzed peroxidation of essential fatty acids—reflect oxidative stress in different neurological disorders including MS. In RRMS it is supposed to be a marker of neurodegeneration with axonal injury, in which oxidative stress is involved [167]. A recent study measured this marker in patients experiencing the first clinical attack suggestive of MS, where increased levels of IsoP in CSF indicated the presence of oxidative stress in the very early course of the disease. This finding strengthens the theory that neurodegeneration occurs at the early onset of the disease [167]. Teunissen et al. found decreased IsoP serum concentration compared with CIS [168].

According to a study, decreased vitamin D-levels might be responsible for the development of MS [169]. Vitamin D-binding protein (DBP) was shown to be more oxidized in remitting and relapsing phases but with increased oxidation rates during relapses. The higher oxidation rate of DBP in remission indicates that some molecular processes are active and not perfectly inhibited in remission [170]. The oxidation of apolipoprotein A-IV increased with the progression of the disease [170].

In one pilot study, consecutive CSF samples from a fulminant MS case were examined. Seven proteins among seventy-eight biomarkers were detected to be elevated in RRMS patients as compared to heathy controls. They were responsible for immune response, blood coagulation, cell proliferation, and adhesion [171]. Further studies are needed to investigate this question with higher case numbers. The use of samples from biobanks could be a possibility to gather data about rare variants.

Despite the remarkable quantity of research in biomarker investigation only a few agents have been precisely validated and utilized in clinical practice [156]. There are difficulties in conferring the results because of differences in research methods, sample collection, and processing, as well as problems in storage and gathering data from published research. These lead to turmoil and hinder validation and efforts at putting biomarkers into use. To solve these problems, BioMS-eu (available on: http://www.bioms.eu) network made a “consensus protocol for the standardization of cerebrospinal fluid collection and biobanking” in 2009 with an update in 2011 [172], for the purpose of standardizing sample collection, elaboration, storage, data acquisition, and processing, together with general database postulation. Obtaining more data and increasing the value of smaller investigations are expected with the use of these guidelines [172].

## 6. Therapeutic Trials

**Figure 2:**
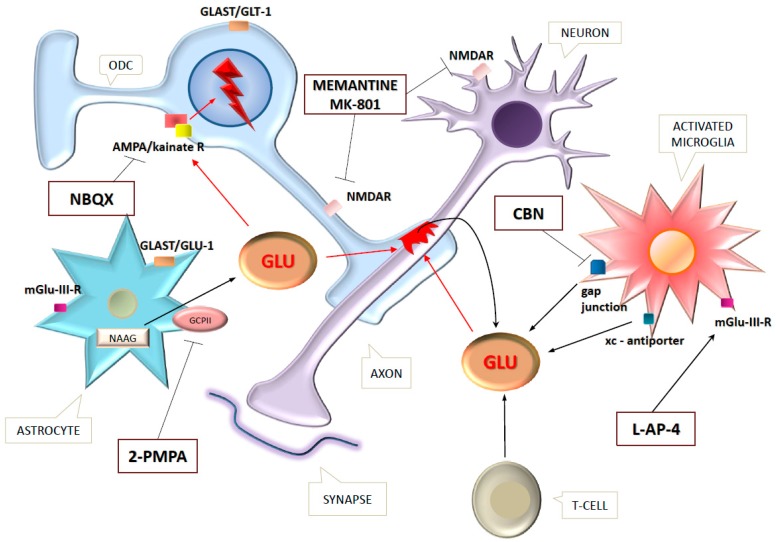
Therapeutic agents and their target points in EAE. The red frames show the site of the damage, the red arrows the route of excitotoxicity, black arrows indicate the direction of a reaction, and T sign mean blockade. Abbreviation: ODC: oligodendrocyte; R: receptor; GLU: glutamate; mGluIII-R: metabotropic glutamate-receptor; NMDAR: *N*-methyl-d-aspartate receptor; GLAST: glutamate-aspartate transporter, in human it is called EAAT1 (excitatory amino acid transporter-1); GLT-1: glial transporter-1, in human it is EAAT2; AMPA: α-amino-3-hydroxy-5-methyl-4-isoxazolepropionicacide; xc-antiporter: system x_c_^−^ cystin/glutamate antiporter; GCPII: Glutamate carboxipeptidase II; NAAG: -acetylaspartylglutamate; CNS: central nervous system.

Despite the newest efficient immunomodulatory therapies, the complexity of MS and lack of further treatment effectiveness have turned attention to the examination of novel therapeutic opportunities. The fundamental and general participation of Glu in ODC death, axon damage, and BBB-dysfunction provides a promising target. Glutamate excitotoxicity is a common pathological reaction for different noxa attacking CNS cells [5], so experimental drugs hold hope for treating not only MS but other severe neurodegenerative disorders like ALS, Alzheimer’s or stroke [73,75,77,78]. In EAE, a great variety of drugs connected to Glu metabolism have been tested. The different targets and drugs are shown in Figure 2.

One of the promising agents is matrine which is a natural alkaloid component of the Radix Sophorae Flaves, formerly used to treat hepatitis-B. It has anti-inflammatory effects and protects against demyelination. In EAE the use of matrine, resulted in Glu downregulation, EAAT overexpression, and decrease in NMDA/AMPA-levels [173].

Ha et al. (2016) [29] examined the impact of an efficient GCPII inhibitor, 2-phosphonomethyl pentanedioic acid (2-PMPA), on EAE mice and detected improvement in disease course and significantly decreased amounts of inflammatory cells infiltrating the CNS. This agent impeded the expression of mGluR1 in CNS and periphery too. Carbenoxolone, a microglia gap junction-blocker prevented neuronal cell death in a dosage-dependent manner and reduced the clinical symptoms of EAE [174].

The inflammatory and excitatory mechanisms which develop simultaneously provide dual targets for treatment. Kanwar et al. (2004) used anti-inflammatory (anti-MAdCAM-1: mucosal addressin cell adhesion molecule-1 monoclonal antibody) and neuroprotective (NBQX: AMPA-antagonist and/or GPE: N-terminal tripeptide of insulin-like growth factor) drugs in combination and found a decrease in clinical symptoms, along with remyelination and ODC viability advancement [175].

There are attempts to use KYNA analogues to treat neurodegenerative disorders [72,76,176,177,178,179,180]. The structural KYNA analogue quinoline carboxamide (laquinimod) slowed the progression of MS and the annual relapse rate in a phase 3 study [71]. Laquinimod may cross the BBB which is an important aspect of this treatment as the endogenous metabolite KYNA does not have this ability. It has great effects on immunoregulation by reducing antigen presentation and offsetting the immune response from Th1 to Th2, amongst others [71].

Luchtman et al. (2016) investigated the effects of immunomodulatory drugs on excitotoxicity in vitro. They found protective impacts of fumarates. Monomethyl-fumarate inhibited Glu release from Th17 cells too [181]. l-2-amino-4-phosphonobutanoate (l-AP-4), a specific agonist of mGluR III, enhanced the amelioration of EAE in Lewis rats [182].

However, the great outcomes seen in animal experiments (disease amelioration, decreased ODC death, and reduced axon injury) could not be observed in human research. i.e., memantine showed promising disease-modifying effects in EAE but its human trial was suspended because of disease progress [30,183]. The reason for this difference is unknown and needs to be investigated. The results of animal models and cell cultures can only be reported with caution, indicating the need for perfecting our own models.

Contrary to other neurodegenerative diseases, changes in mitochondrial function could be discovered early in the disease course of MS. Early consequences of synaptic dysfunction are motor and synaptic fatigability, while late consequences are synaptic loss. Evaluation of the reversible phenotype of patients by distinguishing them from the irreversible helps in identifying relevant patients groups with a therapeutic window for intervention.

Currently we lack medicine that could decrease or completely stop mitochondrial destruction and neurodegeneration. Possible therapeutic approaches target different parts of the mitochondrion (electron transport chain, ATP synthase, ROS). Some promising results from animal models shed light on different molecules. In experimental situations, insertion of *superoxide dismutase 2* gene (*SOD-2*) neutralizes the superoxides, thereby stabilizing the integrity of the axon [184]. Knockout CyPD and p66ShcA significantly decreased axonal damage in EAE [110,185]. Currently two studies have described the defensive property of MitoQ, a synthetic antioxidant, in EAE. MitoQ did not affect inflammation, but decreased the extension of axonal damage [186,187]. Molecules increasing PGC-1a activity have extended neuronal survival in animal models [186,187,188,189,190]. In a recently published study, the molecule brain-derived neurotrophic factor (BDNF), originating from the CNS, may mediate axonal defense in EAE [191,192]. Two studies showed the increased secretion of BDNF as an effect in alemtuzumab and laquinimod intake [193,194]. In EAE, fumaric acid ester that is widely utilized in therapy of psoriasis, may yield neuroprotective effects [195,196]. Two molecules, dimethyl-fumarate and its primary metabolite, monomethyl-fumarate, have been found to increase cellular redox potential, glutathione and ATP levels, along with mitochondrial membrane potential. Thus, along the Nrf2 route those neurons were protected from damaging effects of astrocytes [197]. Nrf2 activates different antioxidants leading to a decrease in neurodegeneration [198].

Talla et al. recently decreased mitochondrial oxidative stress and apoptosis in EAE retina with an intravitreal injection of self-complementary adeno-associated virus containing the *NADH-dehydrogenase type-2 complex I* gene as gene therapy, and managed to decrease axonal loss in the optic neuron [199]. Sirtuins SIRT-3 through SIRT-5 were identified as possible potent neuroprotective factors in demyelinating diseases. In one study Rice et al. found that sirtuins may carry a defensive effect against oxidative stress and excitotoxicity [200].

During the tricarboxylic acid (TCA or Krebs) cycle a significant amount of superoxide species is produced, which enhances neurodegeneration even further. According to several studies, a ketogenic diet helps to maintain ATP levels (by replacing intermediates of the TCA cycle) in the dysfunctional mitochondrial respiratory chain [90,95]. A study describes that beta-hydroxybutyrate attenuates decreased ATP production due to the failure of complex I, thus decreasing neurodegeneration in MS [201]. Biotin is essential for free fatty acid synthesis and energy production. A recently published study showed the daily intake of 300 mg-s of biotine decreased the severity of disability in MS by enhancing axonal remyelinisation [202]. Pyrimidine and its derivatives play an important role in the immune system (cellular adhesion and proliferation etc.). The need for pyrimidine in activated and proliferating lymphocytes is increased in MS, thus, the level of dihydroorotate dehydrogenase (DHODH)-dependent de novo pyrimidine synthesis increases simultaneously. Teriflunomide inhibits DHODH and as such, suppresses the JAK-STAT mediated synthesis and the secretion of proinflammatory cytokines (IL-17 and TNF) [203].

Key elements of therapeutic intervention could be Nrf2, DHODH and the TCA cycle.

While searching for antioxidant therapies, vitamin A and E have thus far been the most promising candidates as modulators in MS for future studies [204]. Modern research made it possible to quantify GABA, GSH, and glutamate together with other metabolites relevant in MS at 7T with high accuracy and reproducibility in a single 1-h session [205]. This technique helps profile the metabolic changes during the disease course to identify potentially relevant targets.

## 7. Conclusions

Glutamate excitotoxicity, mitochondrial dysfunction, and redox disturbances are key features in the pathogenesis of MS. Mitochondrial disturbances, neuroinflammation, and increased oxidative stress are closely related processes which show a correlation with axonal degeneration in MS lesions. The CNS is particularly susceptible to oxidative and nitrosative stress. Among possible antioxidants, vitamin A and E are at present the most promising candidates. Glutamate excitotoxicity can be attributed to an increased Glu release, a deficit in Glu reuptake, and altered function of the enzymes participating in Glu metabolism or glutamate receptors. Kynurenines may influence glutamatergic processes, among these, KYNA might act as a neuroprotective molecule by counteracting glutamate excitotoxicity. The identification of potential novel candidates which may prevent oxidative stress, mitochondrial disturbances or excitotoxicity stands in the focus of research. Another main aim of investigations is the identification of biomarkers for MS, which may promote not only the early diagnosis but also the development of personalized therapy.

## Figures and Tables

**Table 1:** Comparison of Glu and its non-active form, glutamine levels in patient cerebrospinal fluid (CSF) samples with different kinds of disorders. Results compared with controls and peripheral facial nerve palsy patients are significant * *p* < 0.05. (Data of Stover et al., 1997 [23]).

Patient Groups	Glutamate Levels in CSF (µM)	Glutamine Levels in CSF (µM)
Controls, *n* = 20	1.3 ± 0.1	574 ± 25
Facial palsy, *n* = 5	1.0 ± 0.1	570 ± 54
MS (non-active disease), *n* = 14	1.2 ± 0.1	467 ± 47
MS (active disease), *n* = 21	3.3 ± 0.3 *	528 ± 22
Meningitis, *n* = 14	2.8 ± 0.2 *	587 ± 35
Myelopathy, *n* = 15	3.1 ± 0.3 *	597 ± 54
Stroke, *n* = 8	2.2 ± 0.2 *	655 ± 31
NPH, *n* = 6	1.7 ± 0.2 *	615 ± 48
Epilepsy, *n* = 4	5.0 ± 1.8 *	629 ± 84

**Table 2:** Glu levels in CNS at different stages of disease and control (measured from patient CSF collected by LP) Data of Sarchielli et al., 2003 [24].

Groups	Glutamate Level (Mean ± SEM, mg/dL)	Significance
Control subjects, *n* = 20	0.050 ± 0.017	NA
RRMS patients (stable phase), *n* = 25	0.080 ± 0.031	Vs. control subjects, *p* < 0.007 Vs. patients with SPMS, *p* = 0.09 Vs. control subjects, *p* = 0.013 Vs. patients without Gd+ lesion, *p* < 0.001 Vs. control subjects, *p* = 0.08 Vs. patients with RRMS assessed during relapse, *p* < 0.001
with Gd+ lesion on MRI, *n* = 14	1.103 ± 0.024	
without Gd+ lesion on MRI, *n* = 11	0.053 ± 0.017	
RRMS patients (active disorder- sample gathered after 72 h of onset) *n* = 30	0.103 ± 0.033	Vs. control subjects, *p* < 0.001 Vs. patients with RRMS during a stable phase, *p* < 0.001
SPMS subjects *n* = 25	0.073 ± 0.024	Vs. control subjects, *p* < 0.01 Vs. patients during stable phase, *p* = 0.13 Vs. patients with RRMS during relapse, *p* < 0.003 Vs. control subjects, *p* = 0.16 Vs. patients with SPMS with at least 1 point increase in EDSS for the last 6 month, *p* < 0.001 Vs. control subjects, *p* < 0.001 Vs. patients with RRMS during relapse, *p* = 0.04
SPMS patients with no EDSS score increasing for the past 6 months, *n* = 13	0.062 ± 0.024	
SPMS patients, whose EDSS score increased at least 1 point for the past 6 months, *n* = 12	0.103 ± 0.014	

**Table 3:** Causes of elevated extracellular Glu levels in CNS in MS/EAE.

**1.** Increased Glu-expression
a. Activated microglia/ma, leukocytes [5,35]—emission channels are:
Gap-junction-like hemipores [36] System x_c_^−^ antiporter [37,38]
b. Astrocytes [4]—causes
Emission via diff. channels (Ca^2+^-dep. and indep., for e. system x_c_^−^) [4] EAAT inversion [4]—direction of Glu-flow changes mGlu-R: Glu binds to it and enhances its own release [39] TNFR1 receptor: TNF-α binds to it and invokes Glu release [40]
c. Demyelinated axons [41]:
i.e., ectopic distribution of Ca^2+^ channels—Ca^2+^ influx invokes Glu-release [41]
**2.** Decreased Glu-reuptake (dysfunction of EAATs)
**3.** Defects of enzymes involved in Glu homeostasis:
Decreased glutamine synthetase-, decreased glutamate-dehydrogenase activity [6,20,34] Increased glutaminase activity (microglia) [21]
**4.** Glutamate receptor overexpression [4,42]
Receptors: ionotropic: NMDA, AMPA, kainite; metabotropic: mGluR-I, mGluR-II, mGluR-III groups Synaptic cleft: concentrations of Glu, GABA and other neurotransmitters alter if synthesis, release, degradation or reuptake changes—in MS/EAE, all of these processes are involved and changes in expression of neurotransmissional receptors [34] Proinflammatory cytokines (TNF-α, IL-β) increase Glu neurotransmission and decrease GABA synaptic signaling [34] Result: altered GABA/Glu neurotransmission with Glu overstimulation and excitotoxicity [34]

**Table 4:** Glu transporters in human and mammals and their occurrence in CNS cells.

Transporter (Human)	Transporter (Mammals)	Occurrence (Cell)
EAAT1	GLAST	Astrocyte, ODC, microglia
EAAT2	GLT-1	Astrocyte, ODC
EAAT3	EAAC1	Neuron (somatodendritic), astrocyte (low)
EAAT4	EAAT4	Purkinje cell
EAAT5	EAAT5	Müller cell (retina)

EAAT: excitatory amino acid transporter; GLAST: Glu-aspartate transporter; GLT-1: glial transporter-1; EAAC: excitatory amino acid carrier; ODC: oligodendrocyte (adapted from Kostic et al., 2013 [4]).

**Table 5:** Classification of metabotropic Glu receptors.

Groups	Subtypes	Localization
Group I.	mGlu1, mGlu5	Neurons: postsynaptic (excitatory effect) Normal case: somatodendritic OPC In MS/EAE: WM, axons
Group II.	mGlu2, mGlu3	Neurons: presynaptic (inhibitor) In MS/EAE: microglia, astrocyte overexpression
Group III.	mGlu4, mGlu6, mGlu7, mGlu8	Neurons: presynaptic (inhibitor) In MS/EAE: microglia, astrocyte overexpression

mGlu: metabotropic Glu receptor; OPC: oligodendroglia precursor cell; MS: multiple sclerosis; EAE: experimental autoimmune encephalomyelitis (adapted from Kostic, 2013 [4]).

**Table 6:** Enzymatic and nonenzymatic antioxidants.

**Enzymatic Antioxidants**	**Source**	**Properties**
Zn/Cu-SOD	nucleus and cytosol	inhibitor of lipid peroxidation
Mn-SOD	mitochondria	inhibitor of lipid peroxidation
catalase	peroxisome	inhibitor of lipid peroxidation
glutathione peroxidase	mitochondria	inhibitor of lipid peroxidation
glucose-6-phosphate dehydrogenase	mitochondria	inhibitor of lipid peroxidation
**Nonenzymatic Antioxidants**	**Source**	**Properties**
α-tocopherol	intravasal, cell membrane	inhibitor of lipid peroxidation hydrophobic scavenger inhibits the propagation of the chain reaction
carotenoids	intravasal, cell membrane	
glutathione	intravasal, mitochondrial, nuclear	inhibitor of lipid peroxidation hydrophilic scavenger prevents the initiation of radical formation
ascorbic acid	intravasal	inhibitor of lipid peroxidation
ceruloplasmin	intravasal	inhibitor of lipid peroxidation
transferrin	intravasal	inhibitor of lipid peroxidation
uric acid	intravasal	inhibitor of lipid peroxidation
Retinol	intravasal	inhibitor of lipid peroxidation
SH groups	intravasal	inhibitor of lipid peroxidation

SH—sulfhydryl, SOD—superoxide dismutase.

## Abbreviations

AMPA	α-amino-3-hydroxy-5-methyl-4-isoxazolepropionic acid
AMPAR	α-amino-3-hydroxy-5-methyl-4-isoxazolepropionic acid receptor
ATP	adenosine triphosphate
BBB	blood-brain barrier
CHI3L1	chitinase 3-like 1 protein
CIS	clinically isolated syndrome
CNS	central nervous system
CNTF	ciliary neurotrophic factor
CSF	cerebrospinal fluid
Cu/Zn SOD	copper/zinc superoxide dismutase
CyPD	Cyclophylin6 D
DNA	deoxyribonucleic acid
DHODH	dihydroorotate dehydrogenase
EAE	experimental autoimmune encephalomyelitis
EAAC	excitatory amino acid carrier
EAAT	excitatory amino acid transporter
GA	glatiramer-acetate
GFAP	glial fibrillary glial fibrillary acidic protein
Glu	glutamate
GluR	glutamatergic receptor
GLAST	Glu-aspartate transporter
GLT-1	glial transporter-1
GSH	reduced glutathione
GSSG	oxidized glutathione
HHV-6	human herpesvirus 6
IL	interleukin
IsoP	isoprostanes
JAK-STAT	Janus kinase/signal transducers and activators of transcription
KP	kynurenine pathway
KYNA	kynurenic acid
LHON	Leber’s hereditary optic neuropathy
MBP	myelin basic protein
MDA	malondialdehyde
mGluR	metabotropic glutamate receptor
MnSOD	manganese superoxide dismutase
MOG	myelin-oligodendrocyte glycoprotein
MRI	magnetic resonance imaging
mtDNA	mitochondrial DNA
N-CAM	neuronal cell adhesion molecule
NFH	neurofilament heavy chain
NFL	neurofilament light chain
NMDA	*N*-methyl-d-aspartate
NMDAR	*N*-methyl-d-aspartate receptor
NO	nitric-oxide
Nrf2	nuclear factor (erythroid-derived 2)-like 2
OCB	oligoclonal band
ODC	oligodendrocyte
QUIN	quinolinic acid
RNA	ribonucleic acid
RNS	reactive nitrogen species
ROS	reactive oxygen species
RRMS	relapsing-remitting multiple sclerosis
S100B	S100 calcium-binding protein B
SH	sulfhydryl
SOD	superoxide dismutase
SPMS	secondary progressive multiple sclerosis
TCA	tricarboxylic acid
TNF-α	tumor necrosis-alpha
TRY	tryptophan
WM	white matter
